# The improvement path of depression and anxiety among adult women in Shanxi Province, China: a fuzzy-set qualitative comparative analysis

**DOI:** 10.3389/fpubh.2025.1531431

**Published:** 2025-05-15

**Authors:** Dahong Wu, Guangxian Zeng, Jing Cheng, Jie Liu, Sitian Li, Mengxia Qin, Lu He, Qilong Feng

**Affiliations:** ^1^Department of Social Medicine, School of Public Health, Shanxi Medical University, Taiyuan, Shanxi, China; ^2^Community Health and Health Development Research Center, Taiyuan, China; ^3^MOE Key Laboratory of Coal Environmental Pathogenicity and Prevention, Shanxi Medical University, Taiyuan, China; ^4^MOE Key Laboratory of Cellular Physiology, Department of Physiology, Shanxi Medical University, Taiyuan, China

**Keywords:** depression, anxiety, adult women, fuzzy-set qualitative comparative analysis, regression

## Abstract

**Background:**

Depression and anxiety (D&A) are currently recognized as complex and prevalent mental disorders that pose major threats to mental health. Women are more susceptible to D&A than men.

**Methods:**

We collected data from female participants in Shanxi Province between November 2021 and March 2022 through on-site investigations and an online survey. The survey collected information on sociodemographic traits, lifestyle factors, and physical and mental health. The degree of D&A was evaluated using the Center for Epidemiological Studies Depression Scale (CESD-10) and the Generalized Anxiety Disorder Assessment Scale (GAD-7). We assessed the impact of these factors on D&A symptoms among women using regression and fuzzy-set qualitative comparative analysis (fsQCA).

**Results:**

D&A symptoms had many common influencing factors. Regression analysis identified key protective factors against D&A, including better self-rated health (Depression: OR = 0.11, 95% CI = 0.03–0.47; Anxiety: OR = 0.11, 95% CI = 0.02–0.57) and the absence of recent illness (Depression: OR = 0.56, 95% CI = 0.38–0.83; Anxiety: OR = 0.49, 95% CI = 0.35–0.70). Age exhibited marginal protective effects for both conditions (OR = 0.99, 95% CI = 0.98–1.00). In contrast, occupational stress constituted a significant risk factor, substantially increasing the likelihood of depression (OR = 2.66, 95% CI = 1.43–4.96) and anxiety (OR = 2.99, 95% CI = 1.43–4.96). FsQCA analysis did not identify the conditions for ideal mental health (all consistency < 0.9). However, it did identify eight condition configurations predicting mental health (absence of depression symptoms), each achieving consistency ≥0.87. Additionally, two distinct configurations explained resilience to anxiety (consistency ≥0.80). All configurations met fsQCA’s consistency requirements, with self-rated health (present in 10/10 pathways), social support (9/10), and marital status (9/10) playing important roles in most configurations.

**Conclusion:**

Women’s mental health faces significant challenges, with D&A being closely intertwined. FsQCA did not identify any specific condition for the absence of D&A symptoms. However, it revealed multiple pathways to mental well-being, highlighting the need for personalized, multifactorial interventions rather than a one-size-fits-all approach. Regression and fsQCA complement each other, offering unique strengths, and their combined insights should be widely applied to broader research and practice.

## 1 Introduction

Mental health issues are becoming increasingly prominent, and mental disorders are a globally recognized public health challenge (1). Depression and anxiety (D&A) are common psychological disorders in the general population, characterized by persistent sadness, changes in appetite and sleep patterns, diminished concentration, loss of interest in activities, excessive worrying, and restlessness (2). The Global Burden of Disease Study of 2019 reported that D&A disorders were the second and eighth leading causes of disability-adjusted life years, respectively (3). Approximately 3.8% of the global population is affected by depression, including around 5% of adults worldwide (3), and around 4.05% of the population suffers from anxiety disorders (4). D&A pose threats to people’s mental health. Among 12 mental disorders, D&A account for the highest proportions of the global disease burden, at 37.4 and 22.9%, respectively; this burden is increasing annually (3, 5, 6). Moreover, D&A is closely associated with premature death caused by suicide and related illnesses (7, 8). Without preventive measures, D&A will continue to affect individuals, families, and society.

Multiple social pressures and challenges increase women’s susceptibility to D&A. Indeed, women are twice as likely to suffer from D&A, compared to men (9). Similarly, they are more vulnerable to the effects of physiological cycles such as menstruation (10), pregnancy (11), and menopause (12), which are often accompanied by fluctuations in hormone levels that may directly affect women’s emotions and psychological states (13). Moreover, traditional gender roles and societal expectations placed on women (14), such as balancing family and career responsibilities, impose additional psychological burdens on women. At the societal level, women may face discrimination, violence (15), and unfair treatment (16), which can negatively affect their mental health.

During the COVID-19 pandemic, women often bore the dual responsibilities of caregiving and managing household finances. Lockdowns and restrictive measures forced many women to face economic problems, such as unemployment, reduced income, or job instability, further exacerbating their psychological burden (17). Women’s D&A issues not only lead to a decline in their mental health but may also negatively affect family dynamics (18), child development (19), and work performance (20). Thus, understanding and addressing women’s D&A issues are not only a matter of individual healthcare but also a matter of concern for society’s overall mental health. It is necessary to identify the risk factors for women’s mental health and explore effective intervention measures.

A substantial proportion of previous literature examining factors influencing mental health has concentrated on regression modeling, exploring unidirectional linear relationships and causal symmetry under the assumption that independent variables are independent. However, extant research exhibits two critical methodological constraints. First, conventional approaches inadequately capture interaction effects and combinatorial pathways among multiple variables. Second, prior studies have yet to systematically identify divergent conditional configurations that lead to equivalent psychological outcomes — a manifestation of multiple conjunctural causation that epitomizes the complexity of psychosocial phenomena. To address these dual gaps in methodological capability and theoretical conceptualization, this study innovatively employs fuzzy-set qualitative comparative analysis (fsQCA), an analytical approach prevalent in management science. Grounded in Boolean logic, fsQCA enables the identification of causal condition sets associated with outcomes through configurational analysis. This methodology not only elucidates non-linear relationships between multivariate predictors and outcomes but also reveals equifinal pathways whereby distinct antecedent combinations converge on identical psychological manifestations, thereby rectifying inherent limitations of conventional regression paradigms (21, 22).

First, we conducted a binary logistic regression analysis to scrutinize the factors influencing the levels of D&A among adult women in Shanxi Province. Subsequently, we selected and incorporated representative variables from demographics, lifestyle, physical health, and social support into the fsQCA model to identify different configurations conducive to psychological well-being. Our primary objective was to systematically explore how conditional configurations of multiple risk factors jointly shape D&A symptoms in women, particularly focusing on nonlinear synergistic interactions and equifinal pathways. Through systematic comparative analysis with conventional regression results, we aimed to validate the complementary utility of configurational approaches in mental health research. Furthermore, we sought to leverage these differentiated configurations to provide empirical insights for identifying subgroup-specific high-risk populations and to inform the future development of tailored mental health intervention strategies, thereby offering a novel analytical framework to support precision mental health research. This will positively affect women’s overall well-being and quality of life.

## 2 Methods

This study collected data from November 2021 to March 2022 using a multi-stage sampling method. To calculate the sample size, we utilized the following parameters per the standard formula for cross-sectional studies:


$$n=\frac{Z^{2}\times p(1-p)}{E^{2}}$$

Z = 1.96 (95% confidence level),

*p* = 0.313 (estimated prevalence of anxiety) (23),

E = 0.025 (margin of error),

Since we used multi-stage sampling with a design effect (DEFF) of 2 (24), the final required sample size was:


$$n_{\text{final}}=n\times \text{DEFF}=1321.7\times 2\approx 2643$$

The base sample size was calculated to be 2,643 (21, 22). To account for potential data loss or outliers, we increased the sample size by 10%, resulting in a final target of 2,908.

The province of Shanxi was divided into four regions based on geographical distribution and economic conditions. Two cities/counties were randomly selected from each region, and the sample size was allocated according to the population proportion in each region. Within each selected city or county, streets and townships were randomly chosen for the survey based on population size. The study included female residents of Shanxi Province who had lived there for at least one year, were proficient in Chinese, and were willing to provide informed consent for voluntary participation. Those who had resided in Shanxi Province for less than one year, were under the age of 18 years, had cognitive or language barriers preventing them from understanding or completing the survey, or were unwilling to cooperate or provide informed consent were excluded. The survey (see Supplementary file 1) comprised a total of 135 questions and covered multiple aspects, including sociodemographic traits, lifestyle, social support, mental health status and knowledge, attitudes, and behaviors related to gynecological diseases (25). We did not collect personal identity information to protect the participants’ privacy; we distributed 3,298 surveys, of which 3,063 valid responses were collected, yielding a response rate of 92.87%.

### 2.1 Measurements

#### 2.1.1 Sociodemographic characteristics

The sociodemographic characteristics assessed included age, place of residence, education level, marital status, and income.

#### 2.1.2 Lifestyle

Lifestyle factors included smoking, alcohol consumption, sleep duration, frequency of physical exercise, and occupational stress.

#### 2.1.3 Physical health

Physical health indicators included self-rated health, chronic diseases, and illness within the past two weeks.

#### 2.1.4 Social support

The Social Support Rating Scale (SSRS) comprises ten items categorized into three dimensions (26, 27): objective support, subjective support, and support utilization. Items 1–4 and 8–10 are rated on a 4-point Likert scale ranging from 1 (*never*) to 4 (*always*). Item 5 consists of five parts, each rated on the same 4-point scale. For items 6 and 7, we assigned a score of 0 if the response was “no source” and 1 if the response was “the source exists.” The SSRS score ranges from 12 to 66, with higher scores indicating better social support. We further categorized social support levels into three groups: low (≤22), medium (23–44), and high (≥45).

#### 2.1.5 Assessment of D&A symptoms

We assessed depressive symptoms using the Center for Epidemiological Studies Depression Scale (CESD-10), a self-report questionnaire that measures the frequency of each depressive symptom occurring in the past week. It consists of ten items, each rated on a 4-point Likert scale ranging from 0 (*rarely*) to 3 (*always*), with a threshold of ten suggesting possible depression (28). We assessed generalized anxiety levels using the Generalized Anxiety Disorder Assessment Scale (GAD-7) (29), a self-administered questionnaire that serves both as a screening tool and a measure of severity for individuals with generalized anxiety disorders (30). We summed responses on the 4-level scale to obtain scores ranging from 0 to 21, categorizing different anxiety levels as normal (0–4), mild (5–9), moderate (10–14), or severe (15–21).

### 2.2 Data analysis

We collected and double-entered the data into EpiData 3.1 software, creating a database of valid survey responses. Prior to analysis, we checked the accuracy and consistency of the data. Of the initial 3,298 respondents, we identified 235 invalid records with missing survey data and removed them.

We analyzed the data using R version 4.3.0, IBM SPSS 27.0, and fsQCA 4.1. We used cut-off values from the CESD-10 and GAD-7 to determine the overall rates of D&A in the population. We dichotomized depressive symptoms using a cut-off value of 10. Simultaneously, we merged “mild anxiety,” “moderate anxiety,” and “severe anxiety” with the anxiety category, and retained the minimal anxiety levels in the non-anxiety category, forming binary variables.

We presented the respondents’ sociodemographic traits as means and standard deviations for continuous data and percentages for categorical data. To determine the potential relationships between sociodemographic traits, lifestyle, social support, depression, and anxiety levels, we performed a single-factor logistic regression analysis using the autoReg package (31) in R 4.3.0, with D&A binary outcomes as dependent variables for all variables. We used backward elimination (conditional) for the binary logistic regression model to calculate odds ratios (ORs) and 95% confidence intervals (CIs) to investigate the relationships between multiple variables, D&A. We deemed a significance level of *p* < 0.05 to be statistically significant.

#### 2.2.1 FsQCA

Qualitative Comparative Analysis (QCA) includes three main categories: crisp set QCA (csQCA), fuzzy-set QCA (fsQCA), and multi-value QCA (mvQCA) (32). Among these, fsQCA is particularly advantageous because, unlike csQCA and mvQCA, which are primarily suited for categorical data, fsQCA can address degree changes and partial membership issues, making it more versatile for analyzing complex social phenomena. Given the nature of our data, which includes both continuous variables (e.g., age, sleep duration) and multi-point scales (e.g., self-rated health measured on a five-point Likert scale), fsQCA was the most appropriate choice to allow for a nuanced analysis of gradations in conditions and outcomes. Furthermore, fsQCA has been widely adopted in recent empirical research, underscoring its validity and utility in exploring complex causal relationships (33).

FsQCA was conducted using fsQCA 4.1 software, incorporating eight configuration factors: age, marital status, education level, frequency of physical exercise, level of occupational stress, self-rated health, sleep duration, and social support. These conditions have been included in most D&A studies as control variables and play important roles in mental health (34–38). They showed statistical significance in the logistic regression analysis. We aimed to identify combinations of conditions that affect mental health.

First, the raw data were transformed into fuzzy sets and calibrated to a range of 0 to 1 according to the method proposed by Raginand Fiss (39). This was based on theoretical knowledge, research background, and data characteristics to determine three substantive thresholds for each variable: full membership (1), non-membership (0), and crossover point (0.5). Binary variables typically do not require calibration. For continuous variables, we used the original distribution’s 90th, 10th, and 50th percentiles to define the thresholds and crossover points, adjusting the percentiles in skewed distributions as necessary. The values 1, 3, and 5 were used as calibration points for five-point Likert scales. To ensure data integrity and analytical precision, a small constant of 0.001 was added to the data for any calibrated fuzzy-set membership score of 0.5 (40).

Subsequently, we conducted a needs analysis, assuming that conditions or combinations were “necessary” when the consistency score exceeded 0.9 (41). Finally, we performed a truth table operation by manually setting the case frequency and consistency level thresholds. Typically, frequency thresholds are set at 1 or 2; however, owing to the large sample size of 3,063 observations, we set the frequency threshold for this analysis to 10 and the consistency level threshold to the commonly used 0.8 (42). We generated three possible solutions for a comprehensive analysis: parsimonious, complex, and intermediate. Core conditions, appearing in both the parsimonious and intermediate solutions, were identified as strongly influencing the results, whereas peripheral conditions, present only in the intermediate solution, played a supporting role.

## 3 Results

### 3.1 Participants’ sociodemographic traits

In total, we included 3,063 participants in the study. Their average age was 34.7 ± 11.1 years, with 1,955 (63.8%) currently married. Most participants had a bachelor’s degree or higher (59.7%). Approximately 55.6% rated their health as good. The average score on the Social Capital Scale was 40.9 ± 7.0. Table 1 presents the participants’ demographic traits, health status, and lifestyle characteristics.

**Table 1:** Demographics, health status, and lifestyle of study participants.

Type	Variables	levels	x_±s/ N (%)
Sociodemographic characteristics	Age(years)	Mean ± SD	34.7 ± 11.1
	Register	Rural	1,189 (38.8%)
		Urban	1874 (61.2%)
	Marital status	Not in a marital state	1,108 (36.2%)
		In a marital state	1955 (63.8%)
	Education level	Primary School and below	31 (1.0%)
		Junior high school	232 (7.6%)
		Senior high School or Technical Secondary School	324 (10.6%)
		Tertiary	648 (21.2%)
		Bachelor’s degree	1,365 (44.6%)
		Postgraduate and higher	463 (15.1%)
	Average annual household income(CNY)	<10,000	596 (19.5%)
		10,000~	421 (13.7%)
		20,000~	432 (14.1%)
		30,000~	562 (18.3%)
		50,000~	722 (23.6%)
		≥100,000	330 (10.8%)
Health status	Self-rated health	Very poor	11 (0.4%)
		Poor	104 (3.4%)
		Fair	1,245 (40.6%)
		Good	1,272 (41.5%)
		Excellent	431 (14.1%)
	Illness in the past two weeks	Medical units for medical treatment	171 (5.6%)
		Self-administered medication	256 (8.4%)
		Missed work or stayed in bed for over a day due to discomfort.	70 (2.3%)
		No physical discomfort	2,566 (83.8%)
	Chronic disease	No	2,523 (82.4%)
		Yes	540 (17.6%)
Lifestyle	Smoke	No	2,966 (96.8%)
		Yes	97 (3.2%)
	Drink	No	1879 (61.3%)
		Yes	1,184 (38.7%)
	Frequency of physical exercise	Never	182 (5.9%)
		Rarely	1,473 (48.1%)
		Sometimes	1,019 (33.3%)
		Often	339 (11.1%)
		Every day	50 (1.6%)
	Occupational stress	Very low	178 (5.8%)
		Low	179 (5.8%)
		General	1,659 (54.2%)
		High	817 (26.7%)
		Very high	230 (7.5%)
	Sleep duration(hour)	Mean ± SD	7.3 ± 1.0
Social support	Social Support Rating Scale score	Mean ± SD	40.9 ± 7.0
	Levels of Social Support Rating Scale	Low level (≤22)	19 (0.6%)
		Medium level (23–44)	2075 (67.7%)
		High level (≥45)	969 (31.6%)

Continuous variables are presented as mean ± standard deviations (SDs); category variables are presented as n (%).

### 3.2 D&A symptoms

**Figure 1:**
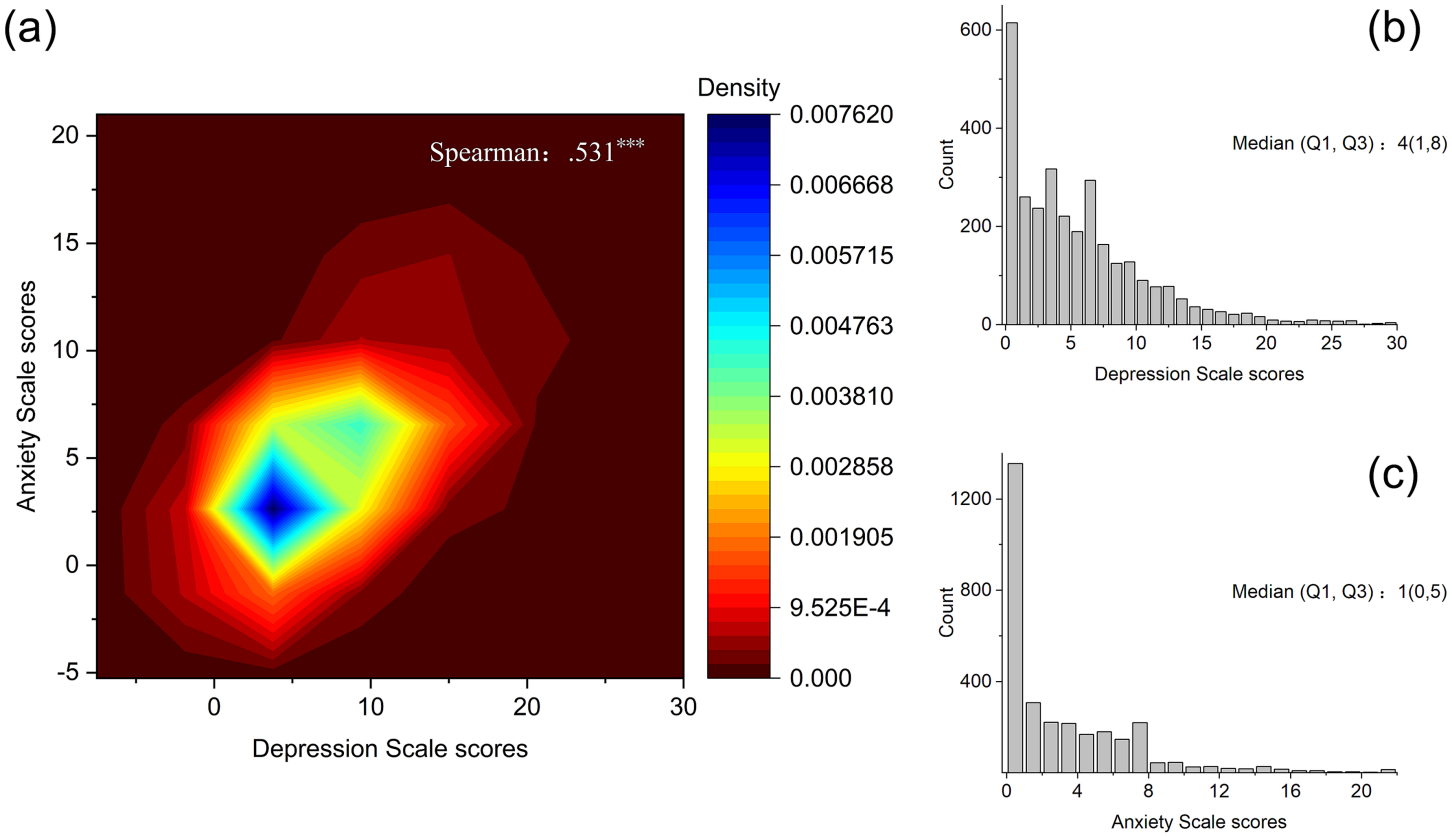
Relationship between depression symptoms and anxiety symptoms. **(a)** Scatter density plot of depression scale scores and anxiety scale scores. **(b)** Histogram of depression scale scores. **(c)** Histogram of Anxiety Scale Scores.

The median score for depressive symptoms on the CESD-10 scale was 4 (1, 8) points, with 510 individuals (16.7%) scoring above the threshold of 10, indicating symptoms of depression. The median GAD-7 score was 1 (0, 5), with 797 individuals exhibiting anxiety symptoms, yielding a screening prevalence of 26.0%. Among them, 343 reported concurrent symptoms of D&A. Spearman’s correlation analysis revealed a moderately positive correlation between the scores on the two scales (not the disorders themselves), which was statistically significant. Figure 1 shows detailed data.

### 3.3 Analysis of the factors influencing depression symptoms

**Figure 2:**
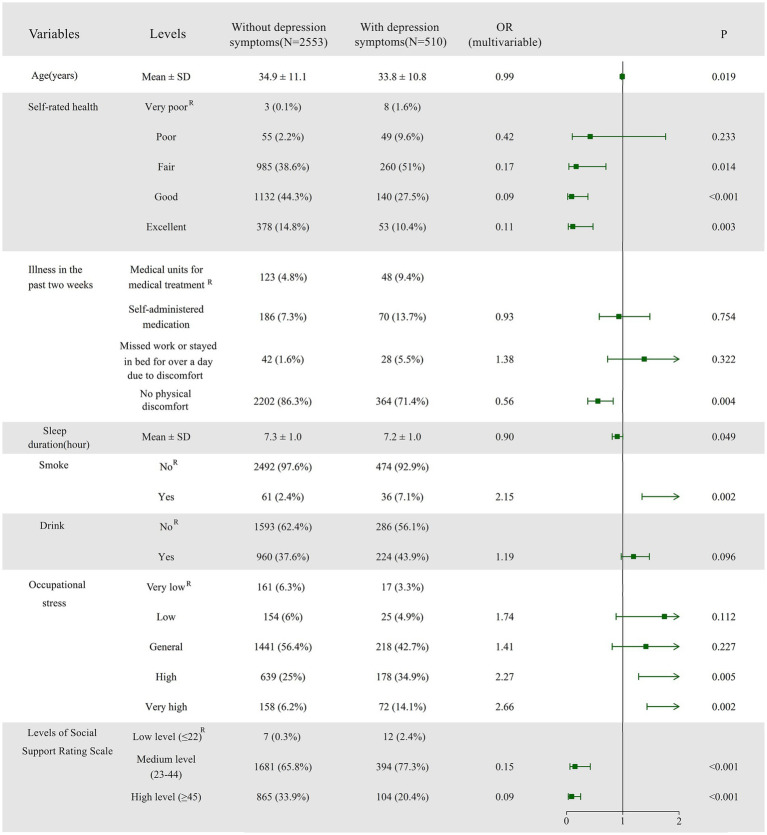
Forest plot of logistic regression analysis identifying factors associated with depression symptoms. The plot presents odds ratios (ORs) with 95% confidence intervals (CIs) for each predictor variable. Additionally, the distribution of influencing factors is displayed, with categorical variables represented as frequencies (%) and continuous variables as means ± standard deviations (SD), providing a comprehensive comparison between the depression and non-depression groups. ^R^ denotes the Reference category, indicating its role as the control group. The coefficients (parameter estimates) of the remaining groups are analyzed in relation to this control group.

Using binary logistic regression analysis, we identified the key factors influencing depression symptoms among adult females. Older adults were less likely to have depressive symptoms (OR = 0.99, 95% CI: 0.98–1.00). Participants who rated their health as fair (OR = 0.17, 95% CI: 0.04–0.70), good (OR = 0.09, 95% CI: 0.02–0.38), or excellent (OR = 0.11, 95% CI: 0.03–0.47) had a lower risk of depression compared to those with poor self-rated health. Individuals who did not experience physical discomfort in the past two weeks were less likely to experience depression compared to those who sought medical care during this period (OR = 0.56, 95% CI: 0.38–0.83). Additionally, longer sleep duration was associated with a protective effect against depression (OR = 0.90, 95% CI: 0.81–1.00). Participants who smoked had a higher level of depression compared to non-smokers (OR = 2.15, 95% CI: 1.34–3.45). Participants experiencing high (OR = 2.27, 95% CI: 1.28–4.01) or very high (OR = 2.66, 95% CI: 1.43–4.96) levels of occupational stress had a greater risk of depression compared to those with low occupational stress, with increasing levels of occupational stress associated with a higher risk of depression. Participants with higher levels of social support had a lower risk of depression compared to those with lower levels of social support, with participants exhibiting medium (OR = 0.15, 95% CI: 0.06–0.42) and high (OR = 0.09, 95% CI: 0.03–0.25) levels of social support, demonstrating a lower risk of depression. To enhance clarity and conciseness, the multivariable logistic regression results are presented as a forest plot (Figure 2), while the detailed regression coefficients and CIs from the final model, along with the results of univariable (single-predictor) logistic regression, are available in Supplementary Table 3.

### 3.4 Analysis of the factors influencing anxiety symptoms

**Figure 3:**
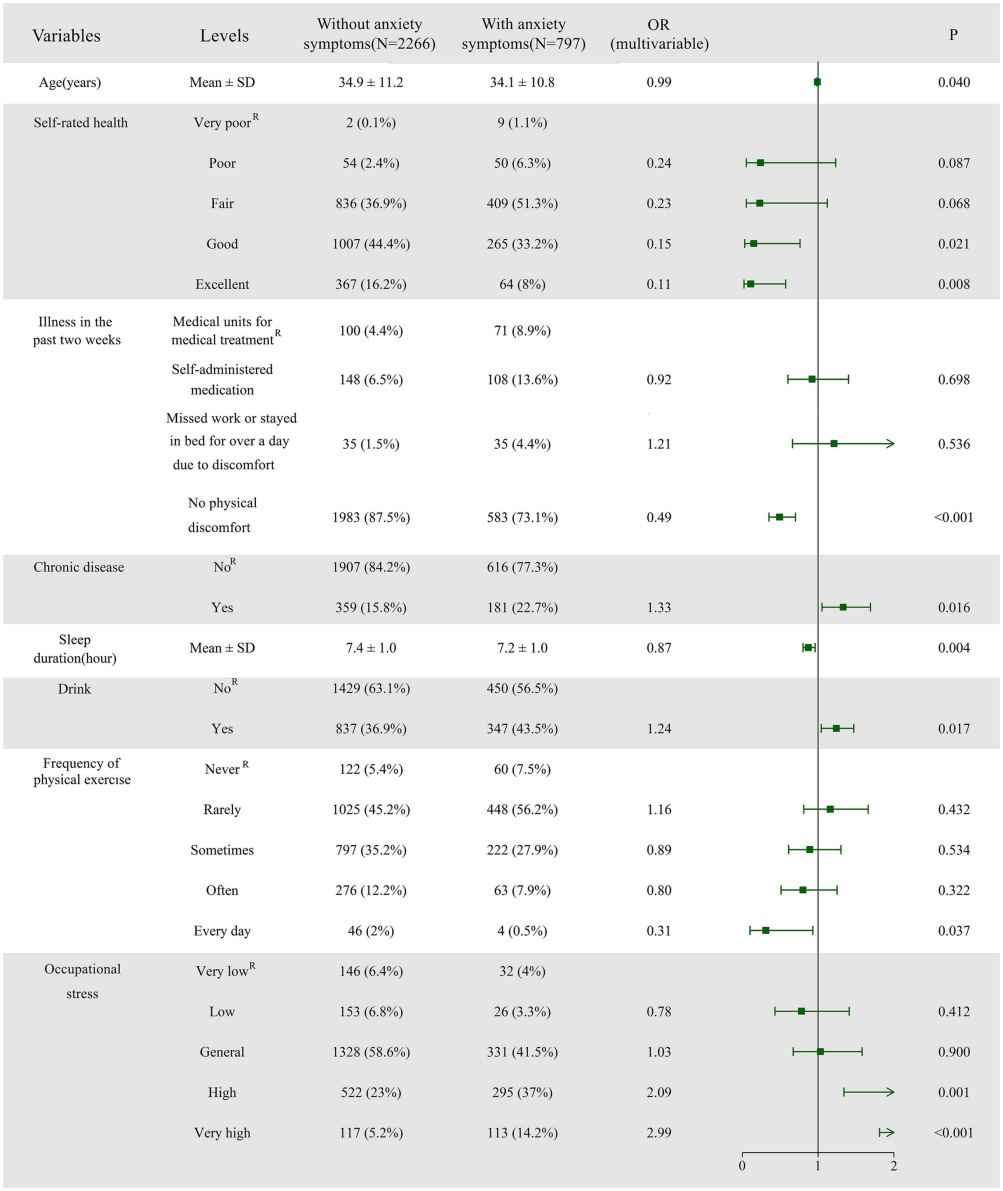
Forest plot of logistic regression analysis identifying factors associated with anxiety. The plot presents odds ratios (ORs) with 95% confidence intervals (CIs) for each predictor variable. Variables with ORs greater than 1 indicate an increased likelihood of anxiety, while those with ORs less than 1 suggest a protective effect. ^R^ denotes the Reference category, indicating its role as the control group. The coefficients (parameter estimates) of the remaining groups are analyzed in relation to this control group.

Figure 3 contains an analysis of the factors influencing anxiety symptoms. The risk of developing anxiety decreased with age: 0.99 (0.98–1.00, *p* = 0.04). We identified better self-rated health—specifically good (OR = 0.15, 95% CI: 0.03–0.76) and excellent (OR = 0.11, 95% CI: 0.02–0.57) health—as a protective factor against anxiety symptoms when compared to very poor self-rated health. Participants who did not experience physical discomfort in the past two weeks were less likely to experience anxiety than those who sought medical care during this period (OR = 0.49, 95% CI: 0.35–0.70). Participants with chronic disease had a greater risk of anxiety (OR = 1.33, 95% CI: 1.05–1.69). Longer sleep duration may have a protective effect against anxiety (OR = 0.87, 95% CI: 0.80–0.96). Alcohol consumption was associated with a higher risk of anxiety compared to non-drinkers (OR = 1.24, 95% CI: 1.04–1.47). Daily physical activity was a protective factor against anxiety compared to no activity (OR = 0.31, 95% CI: 0.10–0.93). We identified high (OR = 2.09, 95% CI: 1.34–3.26) and very high (OR = 2.99, 95% CI: 1.81–4.93) levels of occupational stress as risk factors for anxiety, with increasing levels of occupational stress associated with a higher risk of anxiety. More detailed data are available in Supplementary Table 3.

### 3.5 Modeling FsQCA

#### 3.5.1 Variable selection and calibration

D&A are the most common and significant psychological indicators that often coexist and may influence each other; they can be used as reference indicators to assess mental health status (43). We used depression or anxiety as an outcome variable, with those with no symptoms of depression or anxiety considered psychologically healthy, defined as fully affiliated, and the rest corresponding to non-affiliated thresholds, which did not need to be calibrated.

Simultaneously, we selected significant variables in analyzing factors influencing D&A symptoms, combined with their importance in other studies on D&A, to determine the final variables included in the fsQCA. These variables included age, marital status, education level, physical exercise, occupational stress, self-rated health status, sleep duration, and social support.

The actual score range of the social support scale was 17–64 points, with approximately normally distributed data. We used the original distribution’s 90^th^, 10^th^, and 50^th^ percentiles to define the thresholds and crossover points, resulting in 49, 31, and 41 points, respectively. Age followed a positively skewed distribution, with participants generally younger; thus, we used the 85^th^, 15^th^, and 50^th^ percentiles to define the thresholds and crossover points, resulting in 47, 23, and 33, respectively. We calibrated sleep duration similarly, resulting in 8, 7, and 6. We used an indirectly calibrated method to create a 6-value fuzzy-set for education level. Self-rated health reflects an individual’s subjective evaluation of his/her health status, to some extent indicating real health conditions (44); hence, we consider self-rated health as a variable reflecting physiological health factors. We calibrated the frequency of physical exercise and self-rated health using a 5-point Likert scale, with fully belonging, crossover, and fully non-belonging corresponding to 5, 3, and 1 point(s), respectively. As for occupational stress, where the proportion of very low and low-stress levels was fairly low, and the distribution was skewed, we used the mean value of 3.24 to replace 3 for direct calibration. Marital status, a binary data piece, does not require separate calibration. Table 2 lists the calibrated values of the research variables.

**Table 2:** Assignment of conditioning and calibration parameters for fsQCA.

Condition	Assignment	Fully in	Crossover point	Fully out
Age	Years old	47 [85%]	33 [50%]	23 [15%]
Marital status	1 = Married, 0 = Not married	1	-	0
Education	1 = Primary School and below,2 = Junior high School,3 = Senior high School or Technical Secondary School,4 = Tertiary,5 = Bachelor’s degree,6 = Postgraduate and higher	-	-	-
Physical training	1 = Never, 2 = Rarely, 3 = Sometimes, 4 = Often, 5 = Everyday	5	3	1
Self-rated health	1 = excellent, 2 = very good, 3 = good, 4 = fair, 5 = poor	5	3	1
Occupational stress	1 = Very low, 2 = Low, 3 = General, 4 = High, 5 = Very high	5	3.24 [Mean]	1
Social support	A scale of 17–64 ranging from lowest to highest	49 [90%]	41 [50%]	31 [10%]
Sleep duration	Hour	8 [85%]	7 [50%]	6 [15%]
Depression	1 = Not depressed, 0 = Depressed	1	-	0
Anxiety	1 = Mild/moderate/severe, 0 = Normal	1	-	0

0/1 denotes distinct sets requiring no calibration.

Condition education, calibration by indirectly calibrated method: six-valued fuzzy sets.

#### 3.5.2 Needs analysis

Table 3 presents the results of analyzing the conditions required for mental health (i.e., negative screening results for depression or anxiety on the respective scales). We included all present or absent conditions in analyzing needs-based conditions. As shown in Table 3, we did not find any conditions to be necessary (consistency greater than 0.9).

**Table 3:** Analysis of the necessary conditions for mental health.

Condition	Without depression symptoms		Without anxiety symptoms	
	Consistency	Coverage	Consistency	Coverage
Old	0.500	0.846	0.501	0.753
~Old	0.500	0.821	0.499	0.727
Married	0.647	0.845	0.647	0.750
~Married	0.353	0.814	0.353	0.722
Education	0.693	0.835	0.687	0.735
~Education	0.307	0.830	0.313	0.751
Physical training	0.371	0.853	0.379	0.775
~Physical training	0.629	0.822	0.621	0.720
Occupational stress	0.506	0.809	0.491	0.696
~Occupational stress	0.494	0.860	0.509	0.787
Self-rated health	0.701	0.855	0.707	0.765
~Self-rated health	0.299	0.788	0.293	0.685
Sleep duration	0.629	0.847	0.638	0.762
~Sleep duration	0.371	0.812	0.362	0.704
Social support	0.533	0.871	0.539	0.782
~Social support	0.467	0.795	0.461	0.696

The tilde ‘~’ represents negation.

#### 3.5.3 Sufficiency analysis

In the sufficiency analysis, we set the frequency threshold to 10 to ensure that an adequate number of cases would be retained in the analysis. We set the consistency level threshold to the common threshold of 0.8. For D&A, we obtained 13 and two configurations, respectively. To improve the model’s performance, we adjusted the consistency level threshold for depression to 0.85, resulting in eight distinct configurations, including parsimonious, complex, and intermediate solutions, provided in the fsQCA. We defined configuration factors that appeared simultaneously in the parsimonious and intermediate solutions as core conditions that significantly impact the results. The peripheral conditions played a supporting role and appeared only in the intermediate solutions. Table 4 portrays further details.

**Table 4:** Analysis of the mental health configurations.

Condition	Non-depression								Non-anxiety	
	1	2	3	4	5	6	7	8	1	2
Old		⚫			⊗	⚫	⚫	⊗	⚫	⊗
In married	⚫	⚫	⚫	⚫		⚫	⚫	⊗	⚫	⊗
Education	⚫		⚫		⚫	⚫		⚫		⚫
Physical training			⊗					⬤		⬤
Occupational stress	⊗	⊗		⊗				⊗	⊗	⊗
Self-rated health	⚫	⚫	⚫	⚫	⚫	⚫	⚫	⚫	⚫	⚫
Sleep duration	⚫			⚫	⚫		⚫		⚫	⚫
Social support		⚫	⚫	⚫	⚫	⚫	⚫	⚫	⚫	⚫
Consistency	0.885	0.887	0.875	0.894	0.870	0.884	0.893	0.872	0.822	0.803
Raw coverage	0.206	0.215	0.232	0.192	0.215	0.237	0.214	0.065	0.180	0.062
Unique coverage	0.034	0.007	0.010	0.002	0.038	0.006	0.010	0.005	0.180	0.062
Solution consistency	0.874								0.817	
Solution coverage	0.442								0.242	

Solid black circles represent the presence of a condition, while crossed open circles (‘⊗’) indicate the absence or negation of a condition. Blank spaces signify a lack of significance, indicating that the condition score, regardless of magnitude, was not important in that specific configuration related to the outcome. Core or central conditions are represented by large circles, while small circles indicate peripheral or contributing/complementary conditions.

#### 3.5.4 Configurations for resilience against depression

The analysis yielded eight configurations explaining mental health (without depression symptoms), with consistency exceeding 0.87 for each configuration, indicating compliance with the consistency requirements. Among the eight variables used in this study, self-rated health appeared consistently in each configuration in the present state, implying that higher self-rated health is an important condition for resisting depression. Social support appeared in seven configurations, appearing as a core condition. However, alone, it is not sufficient to achieve good mental health because this requires a combination of multiple conditions to achieve. Marital status appeared as a core condition in six configurations, suggesting that it is a key factor influencing depression. Education level—either as part of the configuration or considered “irrelevant”—appeared next, indicating that higher levels of education help in resisting depression. Sleep duration consistently appeared in the four configurations as a core condition, reflecting the importance of adequate sleep in maintaining good mental health. Occupational stress appeared in configurations 1, 2, 4, and 8, with less occupational stress being more conducive to mental health. Age appeared in five configurations with two states, suggesting that different age groups had different configurations that promoted mental health. In other configurations, age is irrelevant, meaning being “old” does not hinder attaining good mental health when combined with other conditions. Similarly, being young alone is not enough to develop good mental health; a combination of multiple conditions is needed to achieve good mental health status. Physical exercise appeared only twice in these configurations, implying that physical exercise may not promote mental health.

#### 3.5.5 Configurations for suppressing anxiety

Table 4 shows the two configurations for resisting anxiety, with consistency exceeding 0.80. The two configurations were Old*Married*–Occupational stress*Self-rated health*Sleep duration*Social support and Old*–Married*Education*Physical training*–Occupational stress*Self-rated health*Sleep duration*Social support, explaining the configurations of participants of different ages resisting anxiety. Occupational stress, sleep duration, and social support are important factors.

## 4 Discussion

Women often face challenges and confusion in their lives due to mental health issues, such as D&A. According to three nationally representative sampling datasets, the overall prevalence rate of positive depression screenings in the Chinese population is 25.7%, with a positive depression screening rate of 29.5% among women (35). In this study, the positive depression screening rate for women was 16.7%, which is lower than the national level, but close to the 17.8% screening prevalence rate of depression among women in the National Health and Nutrition Examination Survey (NHANES) in the US (45). Additionally, the screening prevalence of generalized anxiety among women was 26.0%, which was slightly lower than the prevalence of anxiety symptoms (31.3%) reported in a survey of the general population’s mental health in China during the COVID-19 pandemic (23). Furthermore, we found a significant positive correlation between the scores on the CESD-10 and GAD-7 scales (not the disorders themselves), consistent with the findings of previous studies (46). Additionally, 67.3% of individuals who screened positive for depression also screened positive for generalized anxiety, indicating a high degree of overlap. This comorbidity is consistent with findings in Western populations, where studies have suggested that shared underlying mechanisms, such as dysregulated stress response (47) and cognitive vulnerability, contribute to the high co-occurrence of D&A (48). These results indicate that mental health issues, especially D&A, are more prevalent among women, highlighting the urgency of addressing women’s mental health problems. We explored the factors influencing D&A among women, including sociodemographic traits (age, place of residence, education level, marital status, and income), lifestyle factors (smoking, alcohol consumption, frequency of physical exercise, sleep duration, and occupational stress), physical health (self-rated health, chronic diseases, and illness within the past two weeks), and social support. In addition, we compared the regression and fsQCA methods.

The regression outcomes showed that D&A symptoms share common influencing factors. Higher age, good self-rated health, no illness in the past two weeks, longer sleep duration, and good social support were common protective factors against D&A symptoms among women, whereas occupational stress had a negative impact, in line with the findings of existing research (36, 38, 49, 50). Additionally, smoking is positively correlated with depression symptoms because it can compromise physical health and may exacerbate the development of depression (51). However, we conducted a cross-sectional study, and the proportion of female smokers was only 3.2%. Therefore, the possibility of some depressed women smoking to alleviate psychological stress cannot be ruled out (52), and the directionality of the relationship between smoking and depression symptoms remains uncertain. Similarly, we found a correlation between alcohol consumption and anxiety symptoms, which may align with the Self-Medication Hypothesis, suggesting that individuals use alcohol as a means of alleviating anxiety (53). Moreover, women with chronic diseases are at greater risk of developing anxiety, further demonstrating the interplay between physical and mental health. Univariate analysis showed that a higher frequency of physical exercise was associated with a lower risk of D&A. In addition, multivariate analysis indicated that physical exercise could be a predictive factor of anxiety, confirming the well-established benefits of physical exercise on both physical and mental health (54).

Logistic regression can be used to identify the degree of influence of individual variables on D&A, whereas fsQCA complements logistic regression’s shortcomings by revealing the complex interactions among multiple factors and exploring causal pathways through a combination of different conditions (55). According to the fsQCA results, we did not observe any conditions necessary for promoting mental health (without D&A symptoms). In the sufficiency analysis, we obtained eight and two configurations to explain mental health.

The results of the depression configuration revealed that self-rated health appeared in all configurations as an existing state, indicating its important role in buffering against depression. Research suggests that various factors influence self-rated health, including sleep quality and chronic pain. Poor self-rated health may increase life-related burdens (56), whereas individuals with good self-rated health are more likely to face life with a positive attitude (57). Therefore, it is recommended that community health services strengthen their focus on individuals with poor self-rated health, regularly monitor their health status, and provide them with psychological counseling and health education. Another crucial variable was social support, which appeared in seven configurations as a core condition. Social support refers to the support individuals receive from others (e.g., spouses, family members, friends, or healthcare professionals). Previous studies have shown a significant positive correlation between low levels of social support and D&A (58). Women with low levels of social support may not receive help from others to reduce their negative emotions, leading to the development of D&A (59). Consistently, studies have demonstrated that increasing social support can significantly reduce depressive symptoms, particularly in women, reinforcing the protective role of strong social networks (60). Through community activities and online and offline support groups, enhancing neighborly interactions and strengthening family support education is important to improve family members’ understanding of women’s emotional needs, and help women balance life stress. Additionally, public awareness campaigns should be utilized to raise societal recognition of women’s mental health needs, encouraging women to actively seek support when facing difficulties.

A significant variable is marriage, which is negatively related to depression and has a significant impact on mental health. Numerous studies have shown that marriage positively affects individual mental health. Compared to married individuals, unmarried, divorced, and widowed individuals are more prone to D&A (61). In configurations 5 and 8, being married appears as “irrelevant” and “absent,” respectively, which suggests that modern young women are open-minded and independent, and that marriage is not the only thing that matters to them. However, they can gain satisfaction and a strong sense of self through their efforts and independent living (62). Among the five configurations where age appeared, individuals of different ages had different paths to resist depression, which explains that age and mental health have different outcomes in different studies and that there may be a non-linear relationship between age and depression (63).

Simultaneously, we found that highly educated women tended to have a lower likelihood of developing depression, which can enhance an individual’s ability to cope with adverse emotions by acquiring more knowledge, skills, and thought patterns, improving cognitive and problem-solving abilities, and thereby better coping with challenges in work and life (64). Comparable findings’ studies suggest that higher education is associated with lower depression risk due to increased psychological resilience and economic stability (65). According to previous literature, longer sleep duration is closely related to mental health. Daily sleep duration influences mental health (66), while D&A can affect sleep quality, creating a vicious cycle (67). Therefore, improving sleep quality among women is essential. Women can be encouraged to establish regular sleep patterns and reminded to avoid caffeine and alcohol intake before bedtime, opting for sleep-inducing herbal teas instead.

Additionally, communities can provide sleep education and training in psychological relaxation techniques to enhance overall sleep quality. Occupational stress appears as “non-existent” in the configurations and is negatively correlated with mental health, which is consistent with the findings of previous studies (68). To alleviate occupational stress and promote employee mental health, enterprises can implement flexible work arrangements and leave policies, provide mental health education and stress management training, establish a supportive work environment, foster teamwork and open communication, and regularly conduct employee satisfaction surveys to identify and address stress-related issues timely. This reinforces previous studies on workplace mental health interventions and highlights the need for targeted policies to mitigate stress among working women (69). In our study, physical exercise did not have the expected effect, with most configurations appearing in a state of “irrelevance.” Although many studies have reported the effects of physical exercise on mental health (70), our study suggests that other factors may attenuate these effects. This finding suggests that the relationship between physical activity and mental health may be more context-dependent than previously assumed, warranting further exploration in future research (71).

Interestingly, configurations 7 and 8 for resisting depression symptoms overlapped with configurations for resisting anxiety symptoms, indicating the presence of common factors or conditions. This overlap implies that a certain degree of correlation exists between D&A rather than a completely independent phenomenon. Some configurations that resist depression also achieve the effect of resisting anxiety when specific factors increase. Specifically, adding low occupational stress in configuration 7 and long sleep duration in configuration 8 can produce the effect of resisting anxiety. This indicates that low occupational stress and adequate sleep duration may play important bridging roles between depression and anxiety and that by regulating these factors, dual positive effects on D&A can be obtained.

These findings provide valuable information for developing more effective psychological health interventions and treatment strategies, as well as clues for a deeper understanding of the relationship between D&A. We call on governments around the world to prioritize women’s mental health issues and take effective measures to protect and promote mental health. Mental health is a vital component of overall societal well-being, affecting the stability and harmony of families and communities. It is essential for governments and all sectors of society to work together to create a supportive environment for women, ensuring they have access to the necessary mental health resources and services to enhance their quality of life and overall happiness.

## 5 Limitations

This study has several limitations. First, the sample’s representativeness is limited, as the data were collected exclusively from Shanxi Province. While this region may represent certain areas in northern China, it could restrict the generalizability of the findings. To enhance the generalizability of our results, future studies could collect data from multiple provinces, such as Guangdong, to represent southern urbanization and Zhejiang to reflect private economic development and conduct in-depth regional comparisons to validate the universality of the configuration model. Second, although we employed two methods to explore the multiple causality and asymmetry among variables, limitations may remain in interpreting the complex dynamic relationships between them. Moreover, to improve the interpretability of QCA results, future studies could utilize machine learning techniques like LASSO regression to identify the most predictive variables before conducting QCA and simplify the model by reducing the number of independent variables.

Additionally, combining QCA with panel data methods in a longitudinal design could help capture the temporal evolution of variable configurations over a 3-5-year period, particularly regarding policy cycles. Third, fsQCA can complicate the interpretation of results when an excessive number of independent variables are included, making it challenging to adequately account for all relevant factors. To address this, future research could prioritize variable selection based on theoretical relevance and use additional robustness checks to ensure the reliability of the findings. Finally, the study’s cross-sectional design prevents us from inferring the direction of causal relationships, which may lead to potential misinterpretations of the findings. To address this limitation, future research could adopt mixed-methods approaches, integrating qualitative interviews with case studies of typical configurations to uncover the micro-mechanisms behind statistical relationships.

## 6 Conclusion

Our study reveals that the prevalence of D&A symptoms among adult women in Shanxi Province aligns with national trends in China, with individuals who screened positive for depression also often screening positive for generalized anxiety, highlighting the pressing nature of mental health challenges in this population. Both regression analysis and fsQCA demonstrated a strong interconnection between D&A, with shared influencing factors such as age, self-rated health, and social support. Moreover, fsQCA results indicated that D&A follow similar configurational pathways, emphasizing the necessity of integrated mental health interventions rather than treating these conditions in isolation.

Notably, fsQCA did not identify any necessary condition for the absence of D&A symptoms, suggesting that mental well-being results from the synergistic effects of multiple protective factors rather than isolated variables. This underscores the importance of a comprehensive, multidimensional approach to mental health promotion, where interventions should consider the combined influence of various factors rather than focusing on singular determinants. Furthermore, the identification of multiple distinct pathways to the absence of D&A symptoms indicates that there is no universal solution. Instead, tailored interventions that account for individual and group differences are crucial for effectively mitigating mental health risks.

From a methodological perspective, our findings highlight the complementary strengths of regression analysis and fsQCA. While regression analysis identifies the significance and directionality of individual factors, fsQCA captures complex causal configurations contributing to mental health outcomes. Integrating both approaches provides a more nuanced understanding of mental health determinants and should be considered in future research to develop more targeted and effective intervention strategies.

## Data Availability

The original contributions presented in the study are included in the article/Supplementary material, further inquiries can be directed to the corresponding author/s.
