# Supplementary material for: The improvement path of depression and anxiety among adult women in Shanxi Province, China: a fuzzy-set qualitative comparative analysis
Source: Front Public Health. 2025 May 15;13:1531431. doi: 10.3389/fpubh.2025.1531431 (PMC12119263; doi:10.3389/fpubh.2025.1531431)
Supplement: Supplementary file 3 [file Table_3.DOCX]

**Supplementary** **table** **1.** Logistic regression analysis to identify influences on depression symptoms.

| Variables | Levels | Without depression symptoms  (N=2553) | With depression symptoms  (N=510) | OR  (univariable) | OR  (multivariable) |
| --- | --- | --- | --- | --- | --- |
| Age(years) | Mean ± SD | 34.9 ± 11.1 | 33.8 ± 10.8 | 0.99 (0.98-1.00, *P*=0.046) | 0.99 (0.98-1.00, *P*=0.019) |
| Register | Rural ^R^ | 975 (38.2%) | 214 (42%) |  |  |
|  | Urban | 1578 (61.8%) | 296 (58%) | 0.85 (0.70-1.04, p=0.111) |  |
| Marital status | Not in a marital state ^R^ | 902 (35.3%) | 206 (40.4%) |  |  |
|  | In a marital state | 1651 (64.7%) | 304 (59.6%) | 0.81 (0.66-0.98, *P*=0.030) |  |
| Education level | Primary school and below ^R^ | 21 (0.8%) | 10 (2%) |  |  |
|  | Junior high school | 190 (7.4%) | 42 (8.2%) | 0.46 (0.20-1.06, *P*=0.068) |  |
|  | Senior high school or Technical secondary school | 273 (10.7%) | 51 (10%) | 0.39 (0.17-0.88, *P*=0.024) |  |
|  | Tertiary | 541 (21.2%) | 107 (21%) | 0.42 (0.19-0.91, *P*=0.028) |  |
|  | Bachelor's degree | 1152 (45.1%) | 213 (41.8%) | 0.39 (0.18-0.84, *P*=0.016) |  |
|  | Postgraduate and higher | 376 (14.7%) | 87 (17.1%) | 0.49 (0.22-1.07, *P*=0.073) |  |
| Average annual household income（CNY） | <10,000 ^R^ | 494 (19.3%) | 102 (20%) |  |  |
|  | 10,000~ | 345 (13.5%) | 76 (14.9%) | 1.07 (0.77-1.48, p=0.698) |  |
|  | 20,000~ | 354 (13.9%) | 78 (15.3%) | 1.07 (0.77-1.48, p=0.695) |  |
|  | 30,000~ | 469 (18.4%) | 93 (18.2%) | 0.96 (0.71-1.31, p=0.797) |  |
|  | 50,000~ | 608 (23.8%) | 114 (22.4%) | 0.91 (0.68-1.22, p=0.518) |  |
|  | ≥100,000 | 283 (11.1%) | 47 (9.2%) | 0.80 (0.55-1.17, p=0.255) |  |
| Self-rated health | Very poor ^R^ | 3 (0.1%) | 8 (1.6%) |  |  |
|  | Poor | 55 (2.2%) | 49 (9.6%) | 0.33 (0.08-1.33, *P*=0.120) | 0.42 (0.10-1.76, *P*=0.233) |
|  | Fair | 985 (38.6%) | 260 (51%) | 0.10 (0.03-0.38, *P<*0.001) | 0.17 (0.04-0.70, *P*=0.014) |
|  | Good | 1132 (44.3%) | 140 (27.5%) | 0.05 (0.01-0.18, *P<*0.001) | 0.09 (0.02-0.38, *P<*0.001) |
|  | Excellent | 378 (14.8%) | 53 (10.4%) | 0.05 (0.01-0.20, *P<*0.001) | 0.11 (0.03-0.47, *P*=0.003) |
| Illness in the past two weeks | Medical units for medical treatment ^R^ | 123 (4.8%) | 48 (9.4%) |  |  |
|  | Self-administered medication | 186 (7.3%) | 70 (13.7%) | 0.96 (0.63-1.49, *P*=0.869) | 0.93 (0.58-1.48, *P*=0.754) |
|  | Missed work or stayed in bed for over a day due to discomfort. | 42 (1.6%) | 28 (5.5%) | 1.71 (0.95-3.06, *P*=0.072) | 1.38 (0.73-2.64, *P*=0.322) |
|  | No physical discomfort | 2202 (86.3%) | 364 (71.4%) | 0.42 (0.30-0.60, *P<*0.001) | 0.56 (0.38-0.83, *P*=0.004) |
| Chronic disease | No ^R^ | 2121 (83.1%) | 402 (78.8%) |  |  |
|  | Yes | 432 (16.9%) | 108 (21.2%) | 1.32 (1.04-1.67, *P*=0.022) |  |
| Sleep duration(hour) | Mean ± SD | 7.3 ± 1.0 | 7.2 ± 1.0 | 0.86 (0.78-0.94, *P*=0.002) | 0.90 (0.81-1.00, *P*=0.049) |
| Smoke | No ^R^ | 2492 (97.6%) | 474 (92.9%) |  |  |
|  | Yes | 61 (2.4%) | 36 (7.1%) | 3.10 (2.03-4.74, *P<*0.001) | 2.15 (1.34-3.45, *P*=0.002) |
| Drink | No ^R^ | 1593 (62.4%) | 286 (56.1%) |  |  |
|  | Yes | 960 (37.6%) | 224 (43.9%) | 1.30 (1.07-1.58, *P*=0.008) | 1.19 (0.97-1.47, *P*=0.096) |
| Frequency of physical exercise | Never ^R^ | 139 (5.4%) | 43 (8.4%) |  |  |
|  | Rarely | 1193 (46.7%) | 280 (54.9%) | 0.76 (0.53-1.09, *P*=0.139) |  |
|  | Sometimes | 883 (34.6%) | 136 (26.7%) | 0.50 (0.34-0.73, *P<*0.001) |  |
|  | Often | 292 (11.4%) | 47 (9.2%) | 0.52 (0.33-0.82, *P*=0.005) |  |
|  | Every day | 46 (1.8%) | 4 (0.8%) | 0.28 (0.10-0.83, *P*=0.021) |  |
| Occupational stress | Very low ^R^ | 161 (6.3%) | 17 (3.3%) |  |  |
|  | Low | 154 (6%) | 25 (4.9%) | 1.54 (0.80-2.96, *P*=0.198) | 1.74 (0.88-3.46, *P*=0.112) |
|  | General | 1441 (56.4%) | 218 (42.7%) | 1.43 (0.85-2.41, *P*=0.175) | 1.41 (0.81-2.46, *P*=0.227) |
|  | High | 639 (25%) | 178 (34.9%) | 2.64 (1.56-4.47, *P<*0.001) | 2.27 (1.28-4.01, *P*=0.005) |
|  | Very high | 158 (6.2%) | 72 (14.1%) | 4.32 (2.44-7.65, *P<*0.001) | 2.66 (1.43-4.96, *P*=0.002) |
| Levels of Social Support Rating Scale | Low level (≤22) ^R^ | 7 (0.3%) | 12 (2.4%) |  |  |
|  | Medium level (23-44) | 1681 (65.8%) | 394 (77.3%) | 0.14 (0.05-0.35, *P<*0.001) | 0.15 (0.06-0.42, *P<*0.001) |
|  | High level (≥45) | 865 (33.9%) | 104 (20.4%) | 0.07 (0.03-0.18, *P<*0.001) | 0.09 (0.03-0.25, *P<*0.001) |
| Abbreviations: OR, odds ratio  "Univariable" is the result of a one-way regression analysis; "multivariable" refers to the results of the backward method in binary logistic regression analyses.  ^R^ denotes the Reference category, indicating its role as the control group. The coefficients (parameter estimates) of the remaining groups are analyzed in relation to this control group. | | | | | |

**Supplementary** **Table** **2.** Logistic regression analysis to identify influences on anxiety.

| Variables | Levels | Without anxiety symptoms  (N=2266) | With anxiety symptoms (N=797) | OR  (univariable) | OR  (multivariable) |
| --- | --- | --- | --- | --- | --- |
| Age(years) | Mean ± SD | 34.9 ± 11.2 | 34.1 ± 10.8 | 0.99 (0.99-1.00, *P*=0.068) | 0.99 (0.98-1.00, *P=*0.040) |
| Register | Rural ^R^ | 868 (38.3%) | 321 (40.3%) |  |  |
|  | Urban | 1398 (61.7%) | 476 (59.7%) | 0.92 (0.78-1.09, p=0.326) |  |
| Marital status | Not in a marital state ^R^ | 800 (35.3%) | 308 (38.6%) |  |  |
|  | In a marital state | 1466 (64.7%) | 489 (61.4%) | 0.87 (0.73-1.02, p=0.091) |  |
| Education level | Primary school and below ^R^ | 17 (0.8%) | 14 (1.8%) |  |  |
|  | Junior high school | 175 (7.7%) | 57 (7.2%) | 0.40 (0.18-0.85, *P*=0.018) |  |
|  | Senior high school or Technical secondary school | 254 (11.2%) | 70 (8.8%) | 0.33 (0.16-0.71, *P*=0.004) |  |
|  | Tertiary | 498 (22%) | 150 (18.8%) | 0.37 (0.18-0.76, *P*=0.007) |  |
|  | Bachelor's degree | 997 (44%) | 368 (46.2%) | 0.45 (0.22-0.92, *P=*0.028) |  |
|  | Postgraduate and higher | 325 (14.3%) | 138 (17.3%) | 0.52 (0.25-1.08, *P*=0.077) |  |
| Average annual household income（CNY） | <10,000 ^R^ | 457 (20.2%) | 139 (17.4%) |  |  |
|  | 10,000~ | 308 (13.6%) | 113 (14.2%) | 1.21 (0.91-1.61, p=0.201) |  |
|  | 20,000~ | 316 (13.9%) | 116 (14.6%) | 1.21 (0.91-1.61, p=0.196) |  |
|  | 30,000~ | 404 (17.8%) | 158 (19.8%) | 1.29 (0.99-1.67, p=0.062) |  |
|  | 50,000~ | 534 (23.6%) | 188 (23.6%) | 1.16 (0.90-1.49, p=0.256) |  |
|  | ≥100,000 | 247 (10.9%) | 83 (10.4%) | 1.10 (0.81-1.51, p=0.532) |  |
| Self-rated health | Very poor ^R^ | 2 (0.1%) | 9 (1.1%) |  |  |
|  | Poor | 54 (2.4%) | 50 (6.3%) | 0.21 (0.04-1.00, *P=*0.050) | 0.24 (0.05-1.23, *P=*0.087) |
|  | Fair | 836 (36.9%) | 409 (51.3%) | 0.11 (0.02-0.51, *P=*0.005) | 0.23 (0.05-1.12, *P=*0.068) |
|  | Good | 1007 (44.4%) | 265 (33.2%) | 0.06 (0.01-0.27, *P<*0.001) | 0.15 (0.03-0.76, *P=*0.021) |
|  | Excellent | 367 (16.2%) | 64 (8%) | 0.04 (0.01-0.18, *P<*0.001) | 0.11 (0.02-0.57, *P=*0.008) |
| Illness in the past two weeks | Medical units for medical treatment ^R^ | 100 (4.4%) | 71 (8.9%) |  |  |
|  | Self-administered medication | 148 (6.5%) | 108 (13.6%) | 1.03 (0.69-1.52, *P=*0.891) | 0.92 (0.60-1.40, *P=*0.698) |
|  | Missed work or stayed in bed for over a day due to discomfort. | 35 (1.5%) | 35 (4.4%) | 1.41 (0.81-2.46, *P=*0.230) | 1.21 (0.66-2.22, *P=*0.536) |
|  | No physical discomfort | 1983 (87.5%) | 583 (73.1%) | 0.41 (0.30-0.57, *P*<0.001) | 0.49 (0.35-0.70, *P<*0.001) |
| Chronic disease | No ^R^ | 1907 (84.2%) | 616 (77.3%) |  |  |
|  | Yes | 359 (15.8%) | 181 (22.7%) | 1.56 (1.28-1.91, *P<0.*001) | 1.33 (1.05-1.69, *P=*0.016) |
| Sleep duration(hour) | Mean ± SD | 7.4 ± 1.0 | 7.2 ± 1.0 | 0.82 (0.75-0.89, *P<*0.001) | 0.87 (0.80-0.96, *P=*0.004) |
| Smoke | No ^R^ | 2207 (97.4%) | 759 (95.2%) |  |  |
|  | Yes | 59 (2.6%) | 38 (4.8%) | 1.87 (1.24-2.84, *P=*0.003) |  |
| Drink | No ^R^ | 1429 (63.1%) | 450 (56.5%) |  |  |
|  | Yes | 837 (36.9%) | 347 (43.5%) | 1.32 (1.12-1.55, *P=*0.001) | 1.24 (1.04-1.47, *P*=.017) |
| Frequency of physical exercise | Never ^R^ | 122 (5.4%) | 60 (7.5%) |  |  |
|  | Rarely | 1025 (45.2%) | 448 (56.2%) | 0.89 (0.64-1.23, *P=*0.481) | 1.16 (0.81-1.66, *P=*0.432) |
|  | Sometimes | 797 (35.2%) | 222 (27.9%) | 0.57 (0.40-0.80, *P=*0.001) | 0.89 (0.61-1.30, *P=*0.534) |
|  | Often | 276 (12.2%) | 63 (7.9%) | 0.46 (0.31-0.70, *P<*0.001) | 0.80 (0.51-1.25, *P=*0.322) |
|  | Every day | 46 (2%) | 4 (0.5%) | 0.18 (0.06-0.51, *P=*0.002) | 0.31 (0.10-0.93, *P=*0.037) |
| Occupational stress | Very low ^R^ | 146 (6.4%) | 32 (4%) |  |  |
|  | Low | 153 (6.8%) | 26 (3.3%) | 0.78 (0.44-1.36, *P=*0*.*377) | 0.78 (0.43-1.41, *P=*0.412) |
|  | General | 1328 (58.6%) | 331 (41.5%) | 1.14 (0.76-1.70, *P=*0*.*530) | 1.03 (0.67-1.58, *P=*0.900) |
|  | High | 522 (23%) | 295 (37%) | 2.58 (1.71-3.88, *P<*0.001) | 2.09 (1.34-3.26, *P=*0.001) |
|  | Very high | 117 (5.2%) | 113 (14.2%) | 4.41 (2.78-6.99, *P<*0.001) | 2.99 (1.81-4.93, *P<*0.001) |
| Levels of Social Support Rating Scale | Low level (≤22) ^R^ | 13 (0.6%) | 6 (0.8%) |  |  |
|  | Medium level (23-44) | 1464 (64.6%) | 611 (76.7%) | 0.90 (0.34-2.39, p=0.839) | 1.27 (0.44-3.62, p=0.658) |
|  | High level (≥45) | 789 (34.8%) | 180 (22.6%) | 0.49 (0.19-1.32, p=0.159) | 0.78 (0.27-2.24, p=0.643) |
| Abbreviations: OR, odds ratio  "Univariable" is the result of a one-way regression analysis; "multivariable" refers to the results of the backward method in binary logistic regression analyses.  ^R^ denotes the Reference category, indicating its role as the control group. The coefficients (parameter estimates) of the remaining groups are analyzed in relation to this control group. | | | | | |
